# Neural Code for Ambient Heat Detection in Elaterid Beetles

**DOI:** 10.3389/fnbeh.2020.00001

**Published:** 2020-02-06

**Authors:** Enno Merivee, Anne Must, Karin Nurme, Andrea Di Giulio, Maurizio Muzzi, Ingrid Williams, Marika Mänd

**Affiliations:** ^1^Institute of Agricultural and Environmental Sciences, Estonian University of Life Sciences, Tartu, Estonia; ^2^Department of Science, University of Roma Tre, Rome, Italy

**Keywords:** antennal dome-shaped sensilla, thermoreceptor neuron, bimodal thermohygroreceptor neuron, peripheral spike bursting, insects

## Abstract

Environmental thermal conditions play a major role at all levels of biological organization; however, there is little information on noxious high temperature sensation crucial in behavioral thermoregulation and survival of small ectothermic animals such as insects. So far, a capability to unambiguously encode heat has been demonstrated only for the sensory triad of the spike bursting thermo- and two bimodal hygro-thermoreceptor neurons located in the antennal dome-shaped sensilla (DSS) in a carabid beetle. We used extracellular single sensillum recording in the range of 20–45°C to demonstrate that a similar sensory triad in the elaterid *Agriotes obscurus* also produces high temperature-induced bursty spike trains. Several parameters of the bursts are temperature dependent, allowing the neurons in a certain order to encode different, but partly overlapping ranges of heat up to lethal levels in a graded manner. ISI in a burst is the most useful parameter out of six. Our findings consider spike bursting as a general, fundamental quality of the classical sensory triad of antennal thermo- and hygro-thermoreceptor neurons widespread in many insect groups, being a flexible and reliable mode of coding unfavorably high temperatures. The possible involvement of spike bursting in behavioral thermoregulation of the beetles is discussed. By contrast, the mean firing rate of the neurons in regular and bursty spike trains combined does not carry useful thermal information at the high end of noxious heat.

## Introduction

Environmental thermal conditions play a major role at all levels of biological organization. An increase in body temperature influences biochemical reactions (Hochachka and Somero, 2002) and metabolic processes (Schulte, 2015), leading to physiological and behavioral changes of individual organisms (Chown and Terblanche, 2007; Abram et al., 2017) and ecosystem processes (Brown et al., 2004; Yvon-Durocher et al., 2012). Dependence of life processes on external temperature is most predictable for small ectothermic animals such as insects, which, unlike endotherms, are typically unable to maintain a constant body temperature by homeostasis. A number of abnormalities occur at the cellular level in response to high temperatures above a species-specific range of thermal preference (Denlinger and Yocum, 1998; Neven, 2000; Hochachka and Somero, 2002; Klose and Robertson, 2004; Robertson, 2004). In ground-dwelling insects, the upper limit of this range does not usually exceed 30°C (Fisher et al., 1975; Thiele, 1977; Buse et al., 2001; Guarneri et al., 2003; Challet et al., 2005). Heat stress also has deleterious effects on insect metabolism, respiration, endocrine, and nervous systems, behavior, oviposition, development, and growth (Denlinger and Yocum, 1998; Chown and Terblanche, 2007). Research activity on various effects of temperature on ectothermic organisms has increased recently (Huey et al., 2012; Gilbert et al., 2014; Sunday et al., 2014; Abram et al., 2017; DeLong et al., 2017; Must et al., 2017; Nurme et al., 2018). In part, this has been motivated by the need to understand how individual organisms and ecosystems respond to ongoing global warming when high-temperature trends and daily extremes could become more commonplace (Morak et al., 2013; Stocker et al., 2013; Li et al., 2018). Thus, it is crucial to explain the basic mechanisms by which cold-blooded animals cope with exposure to high temperatures. Data about heat reception in insects is still insufficient (Dhaka et al., 2006; Abram et al., 2017).

Temperature is a critical environmental factor driving the abundance and geographical distribution of ectothermic organisms (Price et al., 2011; Molles and Sher, 2018). To effectively manipulate the behavior and populations of beneficial and pest insects in agricultural lands to boost crop yields, fundamental knowledge on sensory mechanisms of habitat and microhabitat selection, searching behavior, and thermoregulation of these animals is needed. For insects, microclimatic conditions may be quite disadvantageous, if not lethal, should instant and adequate information about their ambient temperature be lacking (Denlinger and Yocum, 1998; Chown and Terblanche, 2007; Abram et al., 2017). Data about heat reception in insects, including agriculturally beneficial predatory carabids and harmful elaterids, are still insufficient (Dhaka et al., 2006; Abram et al., 2017).

Insects rely heavily on thermosensation. Due to their low body weight, heat exchange with the external environment is very rapid. They are especially vulnerable to high-temperature injury (Klose and Robertson, 2004; Chown and Terblanche, 2007; Bowler and Terblanche, 2008). Insects thermoregulate behaviorally to control their body temperature, and to avoid overheating and death (Coggan et al., 2011; Kleckova and Klecka, 2016; Abram et al., 2017; Nurme et al., 2018). Adequate information on ambient temperature is of vital importance to exhibit a proper behavioral response but very little is known of the neural pathways and coding of noxious heat by peripheral thermoreceptor neurons in these arthropods (Dhaka et al., 2006; Tang et al., 2013; Must et al., 2017; Nurme et al., 2018). The sensory cells responsible for detection of external temperature are located in various morphological types of cuticular structures on the insect antennae classified as sensilla coeloconica, basiconica, trichodea, styloconica, capitula, coelocapitula, dome shape sensilla, et cetera (Altner and Prillinger, 1980; Altner and Loftus, 1985; Ruchty et al., 2009; Di Giulio et al., 2012; Nagel and Kleineidam, 2015; Zauli et al., 2016; Must et al., 2017; Nurme et al., 2018; Schneider et al., 2018). In a sensillum, a single thermoreceptor (cold) neuron usually combines with two antagonistically responding hygroreceptor neurons, the moist air and the dry air neuron, respectively, forming a sensory triad. This classical combination of thermo- and hygroreceptor neurons is widespread in various insect taxa including Blattodea, Coleoptera, Hemiptera, Hymenoptera, Lepidoptera, Odonata, Orthoptera, Phasmatodea (Lacher, 1964; Loftus, 1968; Waldow, 1970; Yokohari and Tateda, 1976; Tichy, 1979; Yokohari et al., 1982; Altner and Loftus, 1985; Nishikawa et al., 1985; Chapman, 1998; Shields and Hildebrand, 1999; Piersanti et al., 2011; Must et al., 2017).

So far, temperature-sensitive neurons capable of properly encoding noxious heat in the range of 30–45°C have been demonstrated only in the dome-shaped sensilla (DSS) of two carabid species, *Pterostichus oblongopunctatus* and *Platynus assimilis*, respectively (Must et al., 2010, 2017; Nurme et al., 2015). The DSSs, earlier also incorrectly termed as campaniform sensilla, occur in small numbers on each antennal flagellomere of many carabid (Merivee et al., 2000, 2001, 2002; Di Giulio et al., 2012) and elaterid beetles (Merivee et al., 1997, 1998, 1999; Zauli et al., 2016).

By their response modality and reaction type, the three antennal DSS neurons of carabids and elaterids are classified as the unimodal cold-hot neuron (CHN), the bimodal moist-hot neuron (MHN), and the bimodal dry-hot neuron (DHN), respectively (Nurme et al., 2015; Must et al., 2017). In earlier literature, these neurons are termed as the cold neuron, the moist neuron, and the dry neuron, respectively (Merivee et al., 2003, 2010; Must et al., 2006a,b). They have an amazing and unique property among insects to change their response modality and spike firing mode depending on ambient temperature. At moderate temperatures [20–25 (30)°C], the CHN, MHN, and DHN behave as a typical triad of insect cold, moist, and dry neuron, respectively. The firing rate of the CHN decreases or temporarily stops when confronted with a temperature step increase and vice versa; its spike production phasic-tonically increases in response to a rapid decrease in temperature (Merivee et al., 2003; Must et al., 2010; Nurme et al., 2015, 2018). Stationary mean firing rate of the CHN unequivocally depends on temperature in some carabid species but not in others (Must et al., 2006a,b). The MHN and DHN antagonistically respond to changes in air relative humidity (RH). Firing rate of the MHN increases and that of the DHN decreases with RH increase; in contrast, spiking activity of the MHN decreases and that of the DHN increases with RH decrease (Merivee et al., 2010; Nurme et al., 2018). As temperature increases above 25 (30)°C, however, all the DSS neurons, one by one, start to switch from regular spiking to temperature-dependent spike bursting, and bursting probability of the neurons increases with temperature increase in carabids (Must et al., 2010, 2017) as well as in elaterids (Nurme et al., 2018). By contrast, spike bursting of the CHN, MHN, and DHN never occurs at 20°C even at RH extremes ranging from 5% to 95% in both carabids (Merivee et al., 2010) and elaterids (Nurme et al., 2018), suggesting that burst firing response of the DSS neurons is induced by high temperatures alone. Must et al. (2017) demonstrated in *P. oblongopunctatus*, that several parameters of bursty spike train patterns of the antennal DSS neurons unambiguously depend on temperature and may precisely encode high temperatures up to lethal levels in a graded manner. A great variety of different burst patterns produced by the three DSS neurons may carry much more precise information on ambient heat conditions than mean firing rate of the (regular) spike trains alone. Unfortunately, no comparable data exist on neural coding of high temperatures by the temperature-sensitive neurons in other insects. Even though the three DSS neurons of the elaterid beetle *A. obscurus* also switch from regular spiking to bursting in response to high-temperature exposure (Nurme et al., 2018), no firm information is available on whether and how various burst patterns depend on temperature in these insects.

In this study, we hypothesize that a broad spectrum of high temperature-induced burst firing patterns produced by the classical sensory triad of thermo- and hygroreceptor neurons rather than mean firing rate or bursting probability alone is a general neural code in peripheral heat detection in many insect groups. To test this hypothesis, we show, for the first time, that high temperature-induced, temperature-dependent spike burst patterns of the sensory triad also occur in insect taxa other than Carabidae. Using extracellular single sensillum recording, we measured six parameters of the bursty spike trains produced by the DSS neurons of the dark elaterid beetle *Agriotes obscurus* (Linnaeus, 1758) in the broad range of steady temperatures from 20 to 45°C with 5°C increments. The parameter responses of the CHN, MHN, and DHN to temperature are described by distinct dose–response curves. Spike bursting ability of the neurons is discussed in the context of heat coding and behavioral thermoregulation of the beetles.

*A. obscurus*, the model insect of this study, is a very destructive agricultural pest whose larvae damage a wide range of crops in Central and North Europe and Siberia (Dolin, 1978; Gurjeva, 1979; Parker and Howard, 2001; Blackshaw and Vernon, 2008). Its reproduction period lasts from May to August in the forest zone and from April to June in the steppe zone (Cherepanov, 1957; Gurjeva, 1979). *A. obscurus* serves as a good model for studies on insect thermoreception for several reasons. First, it allows comparison of functioning of peripheral thermoreceptor neurons in morphologically similar antennal sensilla between two large insect groups, carabids and elaterids. Second, *A. obscurus* seldom flies, mostly walking on the ground. It prefers open agricultural grasslands and crop fields (Traugott et al., 2015), where it may encounter dangerously high-temperature zones on the soil surface as an everyday threat. For example, in summer 2018, in southern Estonia, in open sunlit areas, soil surface temperatures above 40°C were recorded on 50 days (personal communication, The Estonian Weather Service^1^). Ground surface temperatures above 45°C were observed on 26 days, and maximum ground surface temperatures attained 50°C. Thus, sensory neurons on the beetle’s antennae, able to encode noxious heat, would be expected.

## Materials and Methods

### Test Beetles

Reproductively immature adults of the elaterid *A. obscurus* overwintering in their pupal cradles in soil were collected in autumn 2015 and 2016. The beetles were placed in plastic containers (18 × 28 × 9 cm) filled with the mixture of moist sand and soil, 30 animals in each, and maintained in a refrigerator at 2–3°C. Five days before the electrophysiological experiments, the beetles were transferred singly into 50-mm plastic Petri dishes provided with a moist filter paper (Whatman International, UK) disc at the bottom and conditioned at 20°C in the Versatile Environmental Test Chamber MLR-35 1H (SANYO Electric, Japan) at 16 h light and 8 h dark (16L:8D) photoperiod. Conditioning of the animals at 20°C was imperative to achieve sufficient electrical contact with neurons located in the antennal DSS and acceptable signal-to-noise ratio. The beetles were not fed but were provided with clean tap water daily. Electrophysiological experiments were conducted with reproductively mature beetles from March to May 2016 and 2017, respectively, i.e., after their cold reactivation.

### SEM Procedure

Antennae were removed from the *A. obscurus* beetle, put overnight into detergent solution, dehydrated with a graded ethanol series (10%–30%–50%–70%–90%–95%–100%), and critical point dried using a CPD 030 Bal-Tech (Baltzers Liechtenstein). Dried samples were positioned on an aluminum stub with a conductive adhesive carbon disc, sputter coated with a thin layer (30 nm) of gold using a K550 (Emithech, Kent, UK) and examined by Dual Beam FIB/SEM FEI Helios Nanolab 600 (Thermo Fisher Scientific, Hillsboro, OR, USA) using the electron column (operating voltage = 5 kV; current = 0.17 nA).

### Electrophysiology

#### Extracellular Single Sensillum Recordings

**Figure 1:**
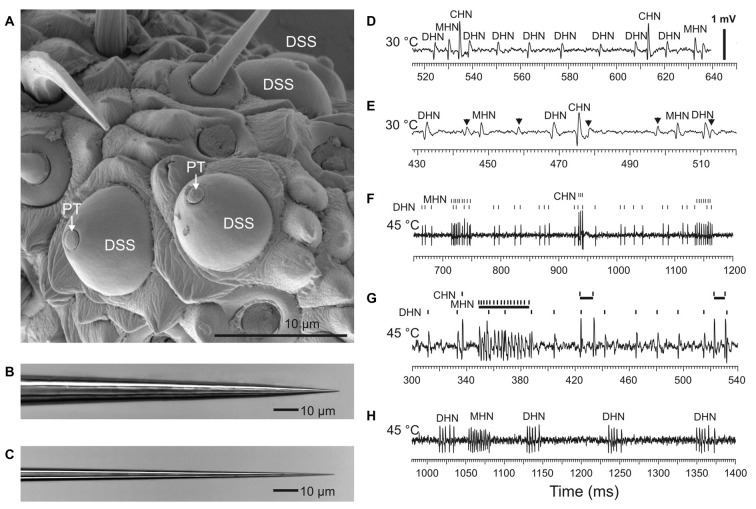
Recording spike firing activity of the sensory neurons innervating antennal dome-shaped sensilla (DSSs) in *A. obscurus*. **(A)** SEM image of a group of DSSs at the tip of the terminal flagellomere favorable for electrophysiological recordings. Note that only a small tip of the sensillum peg (PT) is directly exposed to ambient air. Panels **(B,C)** show tip profile of the tungsten indifferent and recording microelectrode, respectively. The recording electrode used was approximately 0.6–0.8 μm in diameter at a distance of 5 μm from the tip. **(D)** At moderate temperatures up to 30°C, the recording microelectrode typically picked up three different spike waveforms belonging to cold-hot neuron (CHN), moist-hot neuron (MHN), and dry-hot neuron (DHN), respectively. The spikes produced by the CHN were always conspicuously the largest in amplitude. **(E)** In rare cases, the number of spike shapes recorded in a DSS was four, however. The spikes from the “fourth” neuron were always very small, hardly distinguishable among the noise fluctuations in most cases. They originated from some of the adjacent sensilla. In this particular recording, the relatively large spikes from the “fourth” neuron, indicated by arrows, probably arose from the CHN of a neighboring DSS. The spike trains produced by the “fourth” neuron were omitted in this study. **(F)** At high temperatures, the sensory neurons in DSS switched from regular firing to burst firing mode. **(G)** In one and the same DSS, the sensory neurons had a different threshold temperature for spike bursting. In this particular sensillum, at 45°C, spike bursts were only produced by the MHN and CHN while the DHN fired regularly. A sample recording made at 45°C from another DSS demonstrated that high-frequency spike bursts were produced by the MHN and DHN **(H)**. Note that the CHN stopped spike production completely. All the recordings were made from different DSSs.

Our electrophysiological setup used in this study has been thoroughly described earlier (Nurme et al., 2015; Must et al., 2017), so only a brief general characterization is given here. The electrolytically sharpened, indifferent, tungsten microelectrode (Figure 1B) was forced into the base of the flagellum by hand. The recording tungsten microelectrode (Figure 1C) was inserted into the base of a flagellar DSS (Figure 1A) using a DC3KS micromanipulator (Stoelting Co., Wood Dale, IL, USA) under visual control with the electrophysiological microscope Eclipse FN1 (Nikon, Japan). The microscope was equipped with the Nikon ITS-FN1 Physiostage consisting of the X-Y Translator and stainless steel Stage mounted on the Passive Anti-Vibration Science Desk (ThorLabs, UK). All electrophysiological experiments were carried out in a 100 × 120 × 100 cm Faraday Cage FAR01 (ThorLabs, UK). Electrical events recorded in the DSS *via* a custom-made Preamplifier Board (input impedance 10 GΩ; Interspectrum, Estonia) were led to the input of the main amplifier unit ISO-DAM 8A (World Precision Instruments Inc., Sarasota, FL, USA), filtered with a bandwidth set at 10–3,000 Hz, monitored on an oscilloscope screen, and relayed to a computer hard disc for data storage using the 16-bit A/D data acquisition unit Micro 1401-3 (CED, UK) at a sampling rate of 20 kHz and Spike 2 software (CED, UK). Spike-train responses of the sensory neurons were tested at temperatures ranging from 20 to 45°C with 5°C increments. In the first part of the experiment, the mean firing rate of 30 test beetles was recorded and analyzed at each temperature. In the second part of the experiment, the number of test beetles (N) was increased, until bursty spike-train responses from 30 individuals were obtained for analyses in most cases. A couple of exceptions were made, however. Due to the low proportion of bursty spike trains of the DHN and CHN at the low end of noxious heat (below 35°C), *N* for these two exceptional instances was lower, 17 and 19, respectively. The total number of insects used in the experiments was approximately 260.

#### Thermal Stimulation and Temperature Control

Design of our setup for humidity and thermal stimulations of insect antennal sensilla has been described in detail several times earlier (Merivee et al., 2010; Nurme et al., 2015; Must et al., 2017). This time, it would be reasonable to explain the main principles of functioning of the setup in brief. To determine the sensory modality and reaction type of the neurons, thermal and humidity stimuli were presented to the antennal flagellum by two carbon-filtered, preconditioned air streams. For humidity stimulations, both air stream 1 and air stream 2 were preset at the same temperature of 20°C but at different absolute humidity (AH) content, 9 g m^–3^ and 12 g m^–3^, respectively, according to the experimental protocol. Thermal stimulations at different levels of steady temperature by air stream 1 were always performed at constant absolute humidity (9 g m^–3^). The stimulating air stream 1 and air stream 2 at a velocity of 2 m s^−1^ through terminal, aluminum outlet tubes with an inner diameter of 8 mm were pointed at the antennal flagellum of an intact beetle. An electromagnetic air valve (Model 062 4E1; Humphrey Products, Kalamazoo, MI, USA) and a digital timer (Model 4030; Kaiser Fototechnik, Germany) were used to switch rapidly between the two air streams. Both the tested antennal DSSs and a thermocouple junction were located at the intersection of the air stream 1 and air stream 2 with the thermocouple junction at a distance of 1 mm from the tested DSS. The signal from the thermocouple circuit was led to the second input channel of Micro 1401-3 (CED, UK) and saved on a PC hard disc for recording temperature of the stimulating air stream 1 and air stream 2 using Spike 2 software (CED, UK). Custom-made airflow hygrometers (Interspectrum, Estonia) based on the LinPicco™ capacitive humidity analog module A01 (response time <5 s, accuracy < ± 3% RH; Innovative Sensor Technology, Switzerland) were used to measure humidity content of air stream 1 and air stream 2 before the heating units, at 20°C. Required humidity contents were achieved by mixing dried air (5% RH) and moistened air (95% RH) in an appropriate proportion. Signals from the hygrometers were led to the third and fourth input channel of the Micro 1401-3 (CED, UK) and stored on the hard disc for data analysis. The sensory neurons in DSS were allowed to be adapted to each stimulating steady temperature for 2 min before the recording with the duration of 10 s was made.

### Data Management

Spike trains obtained by extracellular single sensillum recordings contained spike waveforms from more than one sensory unit. For extracting spike trains produced by individual DSS neurons, automated spike sorting combined with plenty of automated options for spike waveform analysis (principal component analysis, clustering, collision analysis, spike overdraw, etc.), available in CED Spike 2 software, and visual inspection were used. Sensory modality and reaction type of the neurons (the cold neuron, the moist air neuron, and the dry air neuron) at moderate temperatures in the range of 22–27°C were determined by differences between their mean firing rate responses to rapid step changes in stimulating air RH and temperature (for details, see Nurme et al., 2015, 2018). Response parameters (mean firing rate, burst frequency, the number of spikes in a burst, coefficient of variation of inter-spike intervals, percentage of bursty spike in a spike train, and inter-spike interval in a burst) were extracted from the spike trains with a duration of 10 s and analyzed using CED Spike 2 and MS Excel software. Mean firing rate and burst frequency were calculated as the number of spikes and bursts per second, respectively. An inter-spike interval histogram analysis (Cocatre-Zilgien and Delcomyn, 1992; Bakkum et al., 2014) and visual inspection were used to detect the inter-spike interval threshold for classifying spikes as regular or belonging to a burst (for details, see Must et al., 2010; Nurme et al., 2015).

### Statistical Analyses

All the statistical analysis was performed with the statistical software STATISTICA 11 (StatSoft, USA). Firing rate response of antennal DSS neurons to different levels of steady temperature at constant humidity conditions was analyzed with one-way ANOVA and *post hoc* Tukey test. Kruskal–Wallis test was used to evaluate the effect of temperature on the various bursty spike train parameters of the antennal DSS neurons. All test results were considered statistically significant at *P* < 0.05. The number of tested beetles *N* for each data point was 17–30. In the results of statistical tests, *N* values are higher than 30, however, because the statistical software used summarizes the values of each data point subjected to analysis.

## Results

The external appearance of the cuticular parts of the DSSs of *A. obscurus* is shown in Figure 1A. Usually, these sensilla were located very close together, frequently, 1 or 2 μm from each other. They were composed of a prominent, round cuticular dome and a peg tightly situated deep inside the dome. The small tip of the cuticular peg was directly exposed to ambient air only. Extracellularly recorded spike waveforms from the DSSs belonging to the CHN, MHN, and DHN, respectively, are shown in Figures 1D,E.

### Temperature-Firing Rate Curves of the Antennal DSS Neurons

**Figure 2:**
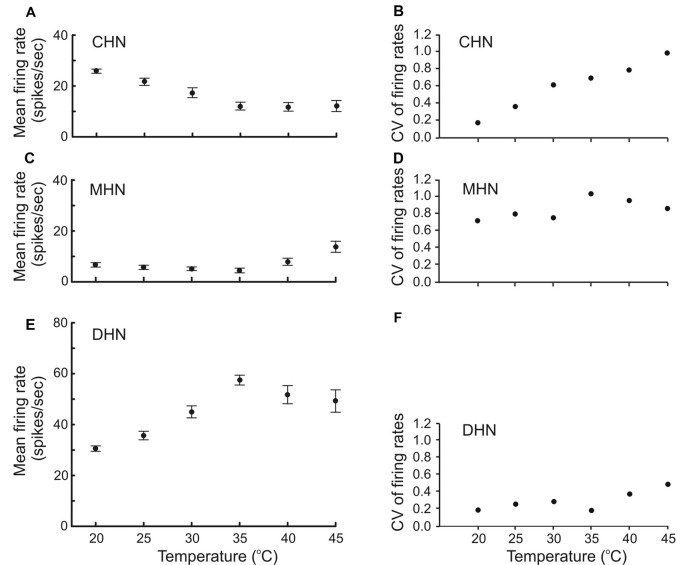
Firing rate response of antennal DSS neurons to different levels of steady temperature **(A,C,E)** accompanied by their coefficent of variation **(B,D,F)**. Both regular spike trains and bursty spike trains of the neurons combined were subjected to the analysis. Recordings were made at constant absolute humidity 9 g m^–3^ condition. Note that mean firing rate of the CHN and DHN did not depend on temperature at the high end of noxious heat ranging from 35 to 45°C **(A,E)**. Also note that response variation of the CHN in different DSS increases with temperature increase **(B)**.Vertical bars show standard errors of the means. CV, coefficient of variation. The number of tested insects for each data point (*N*) = 30.

For the first time in insects, other than carabid beetles, mean firing rate of thermo-sensitive neurons was measured over a broad range of stimulating steady temperatures up to lethal levels ranging from 20 to 45°C. Our experiments with antennal CHN, MHN, and DHN of the elaterid beetle *A. obscurus* showed that these three DSS neurons displayed different stimulus–response curves (Figure 2). At lower temperatures ranging from 20 to 35°C, the mean firing rate of the CHN significantly decreased from 25.8 ± 0.82 to 12.1 ± 1.54 spikes s^−1^ with temperature increase (*F* statistic = 15.63; degree of freedom *df* = 3; mean square MS = 1,037.28; statistical significance *P* = 0.0001; Figure 2A). At temperatures above 35°C, however, this parameter remained at the level of about 12 spikes s^−1^ independent of temperature (*F* = 0.009; *df* = 2; MS = 0.94; *P* = 0.99). A remarkable, nearly linear, 5.7-fold increase from 0.1741 to 0.9845 occurred in coefficient of variation of mean firing rates of the neuron as temperature increased from 20 to 45°C (Figure 2B). Low-level mean firing rates of the MHN varying between 4.5 ± 0.92 and 7.9 ± 1.39 spikes s^−1^ did not significantly depend on temperature in the range of 20–40°C (*F* = 1.89; *df* = 4; MS = 54.02; *P* = 0.11; Figure 2C). A slight increase in the parameter was observed, however, when temperature rose above 40°C (*P* = 0.01). Coefficients of variation of mean firing rates of the MHN across the tested temperatures were relatively high, ranging from 0.7207 to 1.1311, mirroring large variation in spike production between different MHNs (Figure 2D). In contrast, mean firing rates of the DHN significantly increased from 30.6 ± 1.06 to 57.5 ± 1.93 spikes s^−1^ with temperature increase from 20 to 35°C (*F* = 42.15; *df* = 3; MS = 4192.5; *P* = 0.0001), while above 35°C, the parameter lost its sensitivity to temperature (*F* = 1.49; *df* = 2; MS = 533.4; *P* = 0.23; Figure 2E). The calculated coefficients of variation of mean firing rates of the neuron were relatively low, ranging from 0.1838 to 0.4893 with higher values at the high end of tested temperatures (Figure 2F).

### Spike Bursting of the Antennal DSS Neurons at High Temperatures

**Figure 3:**
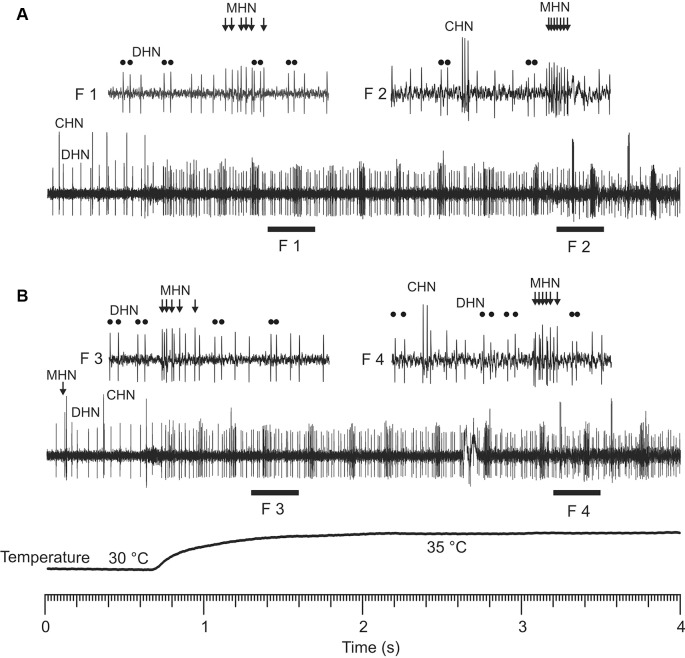
Repeatability of spike burst responses of the DSS neurons in a rapid warming experiment. Note that a particular DSS neuron produced nearly identical bursty spike trains **(A,B)** in response to the same consecutive warming stimulus. Temporal interval between the two stimulations was 10 min. F1–F4 show fragments of the original recordings **(A,B)** in a shorter timescale. High-frequency spike bursts of the CHN were shown in the fragments F2 of **(A)** and F4 of **(B)**, respectively. Double round dots demonstrate bursty spikes of the DHN in the fragments F1–F4, respectively. Bunches of arrows in the fragments F1–F4 indicate bursty spikes generated by the MHN.

**Figure 4:**
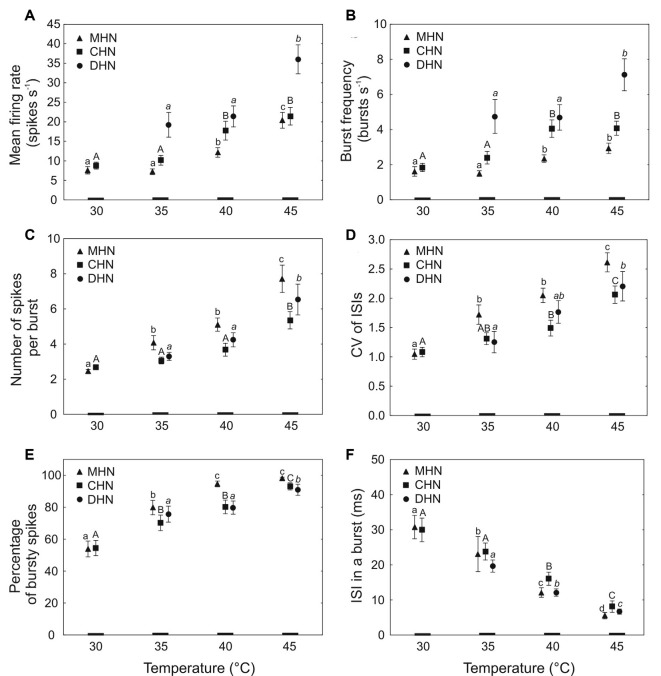
Dependence of bursty spike train parameters (mean ± standard error) of the antennal DSS neurons on temperature **(A–F)**. Different lowercase normal, uppercase normal, and lowercase italic letters show significant differences between the means at *P* < 0.05 (Kruskal–Wallis test) for the MHN, CHN, and DHN, respectively. The number of tested insects (*N*) for each data point = 17–30. CV, coefficient of variation; ISI, inter-spike interval.

At temperatures above 25 (30)°C, all three DSS neurons started to produce bursty spike trains instead of the regular spiking characteristic of the neurons at lower temperatures (Figures 1F–H), but they did not do so simultaneously at the same temperature. Threshold temperature for spike bursting of the neurons both inside of a certain sensillum and between tested sensilla differed. Below 30°C, only few DSS neurons showed burst firing. At higher temperatures, however, their bursting probability increased. Spike bursting response was intrinsic to the DSS neurons only. In the range of 25–35°C, burst firing was never observed in the salt- and sugar-sensitive neurons innervating antennal chaetoid taste bristles of *A. obscurus*. Repeatability of the burst firing responses of the DSS neurons was high. The neurons produced nearly identical bursty spike trains in response to the same consecutive thermal stimulations (Figure 3). In this study, six parameters of the bursty spike trains, mean firing rate, percentage of bursty spikes in a spike train, coefficient of variation of inter-spike intervals, burst frequency, the number of spikes per burst, and inter-spike intervals in a burst, were measured, and their dependence on temperature was analyzed in the range of 30–45°C (Figure 4).

The first four measured response parameters characterize bursty spike trains of the DSS neurons in general. Firing rate reflecting neuronal activity is a commonly used response parameter of sensory cells for encoding external stimuli. In this study, mean firing rate expresses the mean number of spikes the DSS neurons produce at a certain steady temperature in a second (spikes s^−1^). Bursty spike trains frequently contain both spike bursts and solitary, regular spikes. At a certain temperature, percentage of bursty spikes in spike trains produced by the neurons belonging to different DSS also vary due to a considerable variation in their threshold temperature for spike bursting. Thus, the percentage of bursty spikes is an essential and useful response parameter of the DSS neurons to better characterize bursty spike trains they produce in response to heat. The coefficient of variation of inter-spike intervals (CV of ISIs) is an important, but not the only, parameter that helps decide if a particular spike train contains spike bursts or not. CV of ISIs in regular spike trains of the DSS neurons usually remains below 0.5 (Nurme et al., 2018). Higher values of this parameter serve as an indication of probable spike bursts occurrence in a spike train. Spike burst frequency (bursts s^−1^) mirrors the rate of information transmission to the central nervous system *via* burst packages produced by the DSS neurons. The last two parameters, the number of spikes per burst and the inter-spike intervals in a burst, describe the inner structure of the bursts. They allow evaluation of the number of possible burst patterns produced by the three DSS neurons.

#### Dependence of Bursty Spike Train Parameters on Temperature in the CHN

Mean firing rate and burst frequency of the bursty spike trains produced by the CHN responded similarly to different levels of steady temperature. In the narrow range of 35–40°C, a significant, 1.7-fold increase occurred with temperature increase in both mean firing rate (Figure 4A; *P* = 0.02) and burst frequency (Figure 4B; *P* = 0.03). Comparing mean firing rates of the CHN in bursty spike trains (Figure 4A) and in regular and bursty spike trains combined (Figure 2A), it appeared that none of the mean firing rate values was specific only to bursty spike trains. A dynamic range for the third parameter, the number of spikes in a burst, lay at the high end of noxious heat between 40 and 45°C where a 1.5-fold increase of the parameter was observed with temperature increase (Figure 4C; *P* = 0.005). Across the full range of tested temperatures, the coefficient of variation of inter-spike intervals of the neuron significantly increased (fold change 1.9) with temperature increase (*H* = 22.28; *df* = 3; *N* = 108; *P* = 0.0001), whereby in the range of 40–45°C, the slope of the change was considerably steeper than that observed at lower temperatures (Figure 4D). A significant, 1.7-fold increase also occurred in the percentage of bursty spikes when temperature was raised from 30 to 45°C (Figure 4E; *H* = 33.11, *df* = 3, *N* = 108, *P* = 0.00001), although in the narrow, middle range of 35–40°C, the change was not statistically significant (*P* = 0.28). By contrast, the sixth response parameter of the neuron, inter-spike interval in a burst, steeply and nearly linearly decreased (fold change 3.7) with temperature increase from 30 to 45°C (Figure 4F; *H* = 48.42, *df* = 3, *N* = 108, *P* = 0.00001).

#### Dependence of Bursty Spike Train Parameters on Temperature in the MHN

A significant 2.8-fold increase in mean firing rate of the MHN was observed with temperature increase in the range of 35–45°C (Figure 4A; *H* = 29.50, *df* = 2, *N* = 90, *P* = 0.00001). In the same range of temperatures, burst frequency of the neuron increased 1.9-fold with temperature increase (Figure 4B; *H* = 15.41, *df* = 2, *N* = 90, *P* = 0.0005), but at the high end of tested heat, the change was not significant (*P* = 0.89). The third parameter, the number of spikes in a burst, showed a large, 3.3-fold increase (*H* = 59.84, *df* = 3, *N* = 120, *P* = 0.00001) as temperature increased from 30 to 45°C (Figure 4C), although in the middle range of 35–40°C, the 1.3-fold growth of the parameter was not statistically significant (*P* = 0.29). A significant 2.6-fold increase occurred in the coefficient of variation of inter-spike intervals (*H* = 47.22, *df* = 3, *N* = 120, *P* = 0.00001) when temperature was raised from 30 to 45°C (Figure 4D), but again, in the middle range of 35–40°C, the 1.2-fold rise in this response parameter was not statistically significant (*P* = 0.25). A significant 1.8-fold increase with temperature increase was also observed in the percentage of bursty spikes of the spike trains produced by the MHN in the range of 30–40°C (Figure 4E; *H* = 32.22, *df* = 2, *N* = 90, *P* = 0.00001), but then stabilized. At the high end of tested heat ranging from 40 to 45°C, the small increase by 3.8% in this parameter was not statistically significant (*P* = 0.88). Again, compared to other bursty spike train parameters of the neuron, the sixth parameter, inter-spike interval in a burst, showed the largest change. A significant 5.7-fold decrease in this parameter was observed with temperature increase in the range of 30–45°C (Figure 4F; *H* = 63.99, *df* = 3, *N* = 120, *P* = 0.00001).

#### Dependence of Bursty Spike Train Parameters on Temperature in the DHN

Because the spike bursting probability of the DHN was very low below 35°C, its bursty spike train parameters were analyzed in the range of 35–45°C. At the high end of tested heat when temperature was raised from 40 to 45°C, its mean firing rate, burst frequency, and percentage of bursty spikes in a spike train grew 1.7-fold (Figure 4A; *P* = 0.009), 1.5-fold (Figure 4B; *P* = 0.002), and 1.1-fold (Figure 4E; *P* = 0.001), respectively. A significant 2-fold increase in the number of spikes per burst was observed with temperature increase in the range of 35–45°C (Figure 4C; *H* = 7.96, *df* = 2, *N* = 75, *P* = 0.02), but in the lower range of 35–40°C, the little, 1.3-fold growth of the parameter was not statistically significant (*P* > 0.05). A significant, nearly linear, 1.7-fold increase also occurred in the fourth response parameter of the neuron, the coefficient of variation of inter-spike intervals as temperature was raised by 10°C from 35 to 45°C (Figure 4D, *H* = 6.35, *df* = 2, *N* = 75, *P* = 0.04). By contrast, the sixth parameter, inter-spike interval in a burst, showed a significant, steep, and nearly linear, 2.9-fold decrease with temperature increase from 35 to 45°C (Figure 4F; *H* = 37.64, *df* = 2, *N* = 75, *P* = 0.0001).

### Spike Waveform Degradation in the DHN and MHN at the High End of Noxious Heat

**Figure 5:**
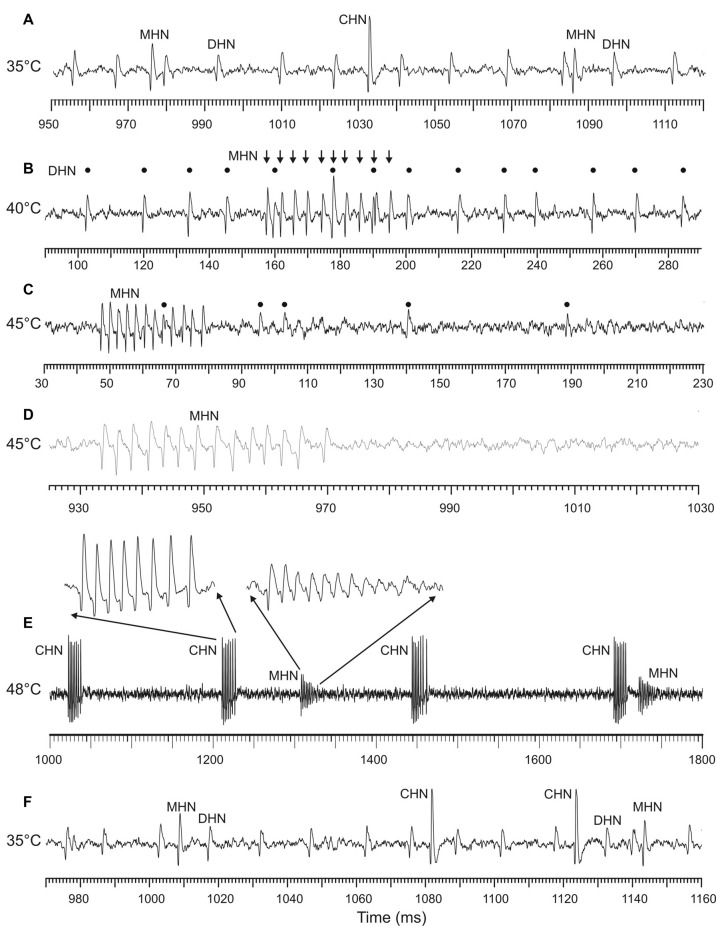
Sample recordings from an antennal DSS, demonstrating degradation of spike waveforms produced by the DHN and MHN at the high end of noxious heat. All the recordings were obtained from the same sensillum. **(A)** At 35°C, in this particular sensillum, all the three neurons fired in a regular manner and displayed normal spike shapes. **(B)** At 40°C, the MHN switched from regular spiking to spike bursting (arrows), the CHN stopped spike production, while the DHN continued regular firing (black dots). When stimulating temperature was raised to 45°C, spike amplitude of the DHN gradually decreased **(C)** and then fully disappeared **(D)** while the MHN continued burst firing by normal spikes **(C,D)**. No spiking activity was observed in the CHN. Note that the spike train fragments **(C,D)** originate from the same 18-s recording. **(E)** When the stimulating temperature was increased further (48°C), spike bursts of the MHN began to degrade also; only the first spike in a burst was of normal amplitude followed by spikes with gradually diminishing amplitude up to the noise level. The DHN remained inactive. By contrast, the CHN became active again, producing high-frequency spike bursts consisting of spikes with normal shape. **(F)** After cooling of the DSS to the initial temperature of 35°C, the DHN and the MHN fully recovered from their high temperature-induced spike shape degradation, demonstrating that the observed changes in spike waveforms were reversible.

Frequently, at temperatures close to 45°C and higher, the ability of the DHN and MHN to produce normal spike waveforms worsened. This was demonstrated by some sample recordings made at different temperatures from one and the same sensillum (Figure 5). Normal spike shapes of the three DSS neurons recorded at 35 and 40°C are shown in Figures 5A,B, respectively. Within a few seconds of exposure to 45°C, spike amplitudes of the DHN diminished down to extinction while the MHN continued to produce normal spikes (Figures 5C,D). When stimulating temperature was raised to 48°C, spike waveform degradation was also observed in the MHN (Figure 5E) while the CHN, after its inactivity at 40 and 45°C (Figures 5B–D), started to generate spike bursts consisting of normal spike waveforms (Figure 5E). Both stopping spike generation in a certain range of high temperatures and spike waveform degradation in the DSS neurons were reversible. When temperature was lowered to a level of 35°C, normal spike waveform of all the three DSS neurons recovered (Figure 5F).

## Discussion

Spike trains of an insect sensory neuron may potentially encode ambient temperatures when an unambiguous relationship between a certain spike train parameter of the neuron and temperature exists. Our electrophysiological experiments with the classical sensory triad of thermo- and hygroreceptor neurons in the antennal DSSs of the elaterid beetle, *A. obscurus*, showed that even though mean firing rate of the unimodal CHN unambiguously depends on temperature in the range of 20–35°C, above that range, the parameter stabilizes at about 10 spikes s^−1^. Similar results with the thermoreceptor (cold) neuron of the triad have been found in the carabid beetle *P. assimilis* (Must et al., 2010). These findings show that mean firing rate of the thermoreceptor (cold) neuron in the classical sensory triad of insects may unambiguously encode moderate ambient temperatures in a graded manner, in some species at least, but it cannot discriminate different levels of noxious heat above 30–35°C. Frequently, the two antagonistic hygroreceptor neurons of the sensory triad are bimodal in terms of their mean firing rate responding to both air RH and temperature (Loftus, 1976; Altner and Prillinger, 1980; Tichy and Loftus, 1990; Tichy and Kallina, 2013; Nurme et al., 2015). Biological relevance of this type of bimodality remains enigmatic, however, because the same mean firing rate values of these neurons cannot encode air RH and temperature at the same time.

Recently, it was discovered that at temperatures of 25–30°C and higher, neurons of the classical sensory triad in insect DSSs start to switch from regular spike firing to spike bursting (Must et al., 2010, 2017; Nurme et al., 2015, 2018). Interestingly, not the cold neuron, but one of the hygroreceptor neurons, the DHN and the MHN in carabid (Nurme et al., 2015) and elaterid beetles (Nurme et al., 2018), respectively, has the lowest threshold temperature for burst firing. This is probably because the outer dendritic segment of the hygroreceptor neurons due to their apical location inside the cuticular peg of the DSSs are more exposed to ambient air compared to that of the CHN (Nurme et al., 2015, 2018; Must et al., 2017).

Several features of spike bursts indicate that they are not abnormalities caused by thermal injury but a normal physiological response of the DSS neurons to high temperatures. The first one is reversibility of the responses. The neurons immediately (within a few milliseconds up to several seconds) switch from regular spike generation to burst firing mode after warming and immediately return to regular spike firing mode after cooling (Must et al., 2010, 2017; Nurme et al., 2015, 2018) with no changes in their spike shape (Nurme et al., 2015, 2018; Must et al., 2017). The next characteristic feature of the neurons is rhythmicity of the bursts in the spike trains they produce. At a constant temperature, the interburst intervals are nearly identical (Must et al., 2010, 2017; Nurme et al., 2015, 2018). Further, at moderate and sublethal temperatures up to 40°C, particular neurons repeatedly generate bursts with nearly a similar number of spikes in a burst and inter-burst intervals (Must et al., 2010, 2017; Nurme et al., 2015, 2018). We underline that in contrast to a variety of sensory systems studied (Kepecs et al., 2002; Gallar et al., 2003; Oswald et al., 2004, 2007; Marsat and Pollack, 2006; Eyherabide et al., 2008), the DSS neurons of carabids and elaterids have a stable and continuous burst train; no temporal information is encoded in the timing of the bursts (Must et al., 2010, 2017; Nurme et al., 2015, 2018). We also emphasize that burst firing responses of the DSS neurons to the same consecutive warming stimulations are highly repeatable. Finally, spike burst generation in response to heat stress is specifically intrinsic to the primary DSS neurons. It is not attributable to the increase of general activity of sensory cells. Even though some central brain cells can produce spike bursts in response to noxious heat (Krahe and Gabbiani, 2004; Hedrick and Waters, 2011; Kim and Connors, 2012), it is not characteristic to other peripheral sensory neurons. For example, spike bursting has never been observed in antennal gustatory neurons of *A. obscurus* and antennal gustatory and olfactory neurons in *Manduca sexta* (Bestmann and Dippold, 1989; Afroz et al., 2013). Thus, the classical triad of insect thermo- and hygroreceptor neurons in DSS sensilla is unique. No other peripheral, presynaptic sensory system is capable of responding to high temperatures in a burst firing mode comparable to that of the DSS neurons of carabids and elaterids.

A spike train parameter of a sensory neuron may unambiguously encode external temperatures in two different ways, in a graded manner that corresponds to the dynamic range of the parameter, and in a level manner corresponding to the specific, unchangeable value of the parameter in a certain range of temperatures. For the first time, our results show that both ways of encoding temperature are possible by bursty spike trains of the antennal DSS neurons in *A. obscurus*. Here, we concentrate on coding of temperatures in a graded manner, however, because it allows more precise discrimination between temperatures. As expected, in this study, we found that in *A. obscurus*, in addition to bursting probability (Nurme et al., 2018), five out of six tested parameters of bursty spike trains of the DSS neurons might potentially encode high temperatures or certain ranges in it from moderate up to lethal levels. Spike burst frequency of the unimodal CHN may encode sublethal heat in a narrow temperature interval between 35 and 40°C just below the critical thermal maximum (CTmax, 39.4°C) of the beetles (Nurme et al., 2018) in a graded manner. In addition, the parameter may roughly discriminate between moderate and lethal heat below 35°C and above 40°C, respectively, in a level manner. Compared to the CHN, the bimodal MHN has the capability to encode sublethal and lethal heat in a broader range between 35 and 45°C in a graded manner. By contrast, spike burst frequency of the bimodal DHN may distinguish lethal high temperatures above CTmax in the range of 40–45°C, which is close to the threshold temperature for total paralysis of the species (41.8°C; Nurme et al., 2018). The second parameter, the number of spikes per burst of the MHN and DHN may distinguish sublethal and lethal high temperatures within a 10°C interval around CTmax of the species (Nurme et al., 2018). In addition, the MHN, by this parameter, may encode moderately high temperatures in the range of 30–35°C, which is just above the threshold temperature for the onset of elevated locomotor activity of the beetles equal to 27.5°C (Nurme et al., 2018). By contrast, the number of spikes in a burst of the CHN may merely encode lethal heat in the narrow range above CTmax of the beetles (Nurme et al., 2018). The next parameter, coefficient of variation of inter-spike intervals of the CHN and MHN may encode high temperatures in a graded manner across the full range of 30–45°C. By contrast, in DHN, the parameter may discriminate temperatures in the 10°C interval of sublethal and lethal heat up to 45°C. The percentage of bursty spikes in spike train produced by the CHN may discriminate high temperatures across the full range of 30–45°C. By contrast, this parameter in the MHN and DHN is capable of encoding high temperatures in a 10°C interval below 40°C and in a 5°C interval above 40°C, respectively. The fifth parameter, inter-spike interval in a burst of all the three DSS neurons, may unambiguously encode high temperatures over the whole range from 30 to 45°C in a graded manner. These results show that in *A. obscurus*, the CHN and DHN function most reliably in the sublethal and lethal ranges of noxious heat while the MHN functions equally well over all ranges from moderate to lethal high temperatures. Our results also show that at high temperature extremes above 45°C, in contrast to the CHN, the ability of the MHN and DHN to produce normal spike waveforms becomes worse or disappears. Thus, the three sensory neurons in antennal DSS of *A. obscurus* seem to encode different ranges of noxious heat almost similar to that observed in carabids (Must et al., 2010, 2017; Nurme et al., 2015). In the carabid *P. oblongopunctatus*, however, the spike bursting DHN reliably encodes moderate and high temperatures in the broad range of 25–45°C while spike bursts of the CHN precisely discriminate sublethal and lethal high temperatures from 35 to 45°C (Must et al., 2017). Our results show that ISI in a burst is the most useful parameter among six parameters. In the parameter, all ISI points of all three receptor neurons are significantly different between adjacent temperatures in the range of 30–45°C.

Reputedly, when ambient air AH is held constant, RH decreases with temperature increase. Thus, temperature and RH influence antennal temperature-sensitive sensilla of the beetles at the same time. Next, we shortly discuss the possible role of ambient RH in spike burst generation in both the DHN and MHN. First, spike bursting of the MHN and DHN never occurs close to 20°C even at RH extremes ranging from 5% to 95% in both carabids (Merivee et al., 2010) and elaterids (Nurme et al., 2018), suggesting that burst firing response of the neurons is induced by high temperatures alone. Second, at a constant AH, mean firing rate in spike trains produced by the DHN below 35°C increases with temperature increase (Nurme et al., 2015, 2018), although in the range of 20–35°C, the neuron is actually insensitive to temperature (Nurme et al., 2015), suggesting that the concurrent RH changes alone drive the firing rate of the DHN. Our results demonstrate that above 35°C, however, mean firing rate in regular and bursty spike trains of the DHN combined stabilizes at about 50 spikes s^−1^, indicating that at the high end of noxious heat, the neuron loses its sensitivity to ambient RH. Thus, it is not likely that ambient RH contributes to the measured parameters of bursty spike trains generated by the DHN at temperatures above 35°C. By contrast, observing mean firing rate of bursty spike trains of the neuron separately, it appears that in the range of 40–45°C, the parameter behaves in a different way, evincing perspicuous increase with temperature increase. Apparently, at 35°C and higher, the proportion of bursty spikes in bursty spike trains strongly prevails compared to solitary spikes. The calculated RH value for AH 9 g m^−3^ at 45°C of 13.7% remains well within the range at which contribution of ambient RH to spike burst generation of both the DHN and MHN is unlikely. Again, temperature alone seems to be responsible for the wide repertoire of bursty spike train patterns that the DHN displays in response to heat. Third, in contrast to the DHN, temperature and air RH oppositely affect mean firing rate in regular and bursty spike trains of the MHN combined (Nurme et al., 2015, 2018). As a result, at constant AH conditions, this parameter does not change in a wide range of temperatures up to sublethal high levels as demonstrated in this study. Bursting probability of the MHN (Nurme et al., 2018) and percentage of bursty spikes in bursty spike trains quickly increase with temperature increase so that at 45°C, almost all the spikes the neuron produces are bursty. In bursty spike trains, however, taken separately, mean firing rate of the neuron shows a strong increase when temperature rises from 35 to 45°C, not explicable with the concurrent RH decrease. To our knowledge, mean firing rate of insect moist neurons increases with air RH increase and vice versa; its spike production decreases with RH decrease (Altner and Loftus, 1985; Chapman, 1998; Merivee et al., 2010; Nurme et al., 2018). These findings suggest that temperature alone drives the great variety of burst patterns the MHN generates in response to noxious heat. Thus, our results show no indications that RH might be involved in spike bursting response of the MHN and DHN.

The functional significance of bursty spike train generation is to increase the reliability of communication between neurons because presynaptic spike bursts of high-frequency firing improve information transmission across unreliable synapses (Lisman, 1997; Krahe and Gabbiani, 2004). Spike bursts evoke higher levels of synaptic neurotransmitter compared to single spikes more sparsely distributed in time. Additionally, bursts might enable more precise information transfer compared to single spikes because spike burst patterns can drive higher neurons more efficiently (Roper et al., 2000; Krahe and Gabbiani, 2004). The central nervous system is able to discriminate between temporal spike train patterns, which have the same mean firing rate but correspond to different temperatures. Thus, in the sensory triad of the antennal DSS neurons of *A. obscurus*, the number of spikes in a burst and the inter-spike interval in a burst are most likely involved in transmission of thermal information on ambient heat. Both of these response parameters contribute to this process. According to this information, it becomes evident that a great variety of possible bursty spike train patterns produced by the DSS neurons of *A. obscurus* may carry much more useful information on ambient heat, and much more reliably transmit it through synapses to the central nervous system compared to information involved in bursting probability of the DSS neurons alone (Nurme et al., 2018). Bursting probability of the neurons does not take into account the level of bursting, i.e., how bursty the bursty spike trains are. When bursting level of a neuron is low, the percentage of bursty spikes is also low, the number of spikes in a burst is two and the inter-spike intervals in a burst are hardly distinguishable from inter-burst intervals or from regular inter-spike intervals in the same spike train (Nurme et al., 2015, 2018). When bursting level of the neuron is high, all the spikes in a spike train are grouped into bursts, the inter-spike intervals in a burst may become extremely short (about 1 ms), and the number of spikes in a burst may reach up to 18 spikes per burst as maximum as demonstrated for the CHN in carabids (Must et al., 2010). In *A. obscurus*, the mean number of spikes in a burst and the inter-spike intervals in a burst produced by the three DSS neurons vary from 2.5 to 8 spikes per burst and from 5.5 to 31 ms, respectively, producing a great variety of different burst firing patterns capable of encoding high temperatures in a graded manner.

Insects are very vulnerable to high-temperature injury. In temperate zones, on sunny summer days, ground surface temperatures in open areas may exceed 40–45°C (personal communication, Estonian Weather Service^2^). In addition, on entering direct sunlight, due to solar IR radiation, a 10-mg insect can heat up by 10°C in only 10 s (Heinrich, 1993). Thus, exposure to ambient heat may be harmful if not quickly lethal for ground-dwelling insects. Heat stress above the upper limit of a species-specific range of thermal preference has deleterious effects at the cellular level, on insect metabolism, respiration, endocrine and nervous systems, behavior, reproduction, development, and growth (Denlinger and Yocum, 1998; Robertson and Money, 2012; Morak et al., 2013). Temperatures above 40°C evoke unusual firing patterns in motor neurons and interneurons and disrupt motoneuronal activity innervating the skeletal muscles (Money et al., 2005; Robertson and Money, 2012), leading to total paralysis and death (Nurme et al., 2018). Hence, insects need to continuously monitor ambient temperature and to respond immediately to deleterious heat when confronted. Our results show that in *A. obscurus*, in the range of 30–45°C, burst frequency of the DSS neurons varies from 1.5 to 7.1 bursts per second with its higher values at the high end of noxious heat. This rate of information transmission to the central nervous system *via* burst packages seems to be high enough, allowing an insect to avoid overheating and death.

A number of studies support the hypothesis that spike bursting is involved in the detection of specific, external signals of danger or behaviorally important stimulus features (Crick, 1984; Sherman, 2001; Swadlow and Gusev, 2001; Kepecs et al., 2002; Krahe and Gabbiani, 2004; Marsat and Pollack, 2006; Eyherabide et al., 2008). In both carabids (Must et al., 2010, 2017) and elaterids (Nurme et al., 2018), the CHN in antennal DSSs is capable of switching between the two firing modes according to the specific features of input temperature. Regular spike trains of the neuron encode moderate temperatures while different patterns of spike bursts are responsible for detecting sublethal and lethal heat. Two other neurons, the MHN and DHN, of the sensory triad in antennal DSSs of carabids and elaterids are bimodal. At moderate temperatures (20°C), they encode ambient air humidity by regular spike trains (Merivee et al., 2010; Nurme et al., 2015, 2018). At higher temperatures, however, the same neurons encode noxious heat *via* various spike burst patterns they produce (Nurme et al., 2015, 2018; Must et al., 2017). Our findings consider spike bursting as a fundamental quality of the thermo- and hygroreceptor neurons in the sensory triad being a flexible and reliable mode of coding unfavorably high temperatures. Its occurrence in two large taxonomic groups of Coleoptera, Carabidae and Elateridae, with more than 40,000 and 10,000 species worldwide (Kromp, 1999; Han et al., 2016), respectively, suggests that it is widespread in many if not all insects with the classical sensory triad of thermo- and hygroreceptor neurons.

Interestingly, polymodality of primary humidity and temperature-sensitive neurons also characterizes other organisms. Particular populations of sensory neurons located on the *Drosophila* antenna respond not only to olfactory stimuli but also to air humidity and temperature (Knecht et al., 2016; Silbering et al., 2016). In *Caenorhabditis elegans*, three types of bimodal temperature-sensitive neurons have been found. In this animal, the primary AWC olfactory neurons also respond to temperature (Biron et al., 2008; Kuhara et al., 2008). The AFD neurons in the head of *C. elegans* respond to both temperature alterations and carbon dioxide (Bretscher et al., 2011; Liu et al., 2012). In addition, a bimodal mechano- and thermosensitive FLP neuron is present in the head of the animal (Liu et al., 2012). Thus, polymodality of sensory neurons seems to be a widespread mechanism through which single neurons broaden their sensory capacity and facilitate multisensory integration. As a result, the same primary receptor neurons can sense distinct sensory modalities and drive behavioral responses through different synaptic outputs (Biron et al., 2008). We speculate that information about ambient air humidity and heat coded by regular and bursty spike trains of the bimodal NHN and DHN, respectively, might be conveyed through different synaptic outputs to respective central neuronal pathways for further processing as well.

Little is known about behavioral discrimination of different levels of noxious heat in insects. Recent behavioral experiments have demonstrated, however, that threshold temperatures for spike bursting of the DSS neurons and onset of elevated locomotor activity of the beetles coincide at 27.5°C (Must et al., 2010; Nurme et al., 2018). From that point up to about 35°C, locomotor activity of the beetles quickly increases with temperature increase, suggesting involvement of the information on ambient heat coded by burst patterns of the three DSS neurons in behavioral thermoregulation. Nurme et al. (2018) also showed that in a slow warming experiment (warming rate 1°C min^−1^; warming range 20–45°C), the beetles of *A. obscurus* reach their maximum locomotor activity level at about 35°C, which they keep up to their critical thermal maximum observed at 39.4°C. Thereafter, locomotor activity of the beetles quickly falls to zero at 41.8°C due to total paralysis and thermoshock. Thus, no correlation exists between spike bursting and locomotor activity of the beetles in the range of 35–45°C. These results suggest that the insects at slow warming or steady temperature conditions do not use thermal information coded by the DSS neurons at the high end of noxious heat. On the other hand, impact of high temperatures on the behavioral performance of animals depends on both the magnitude of temperature change and duration of exposure (Kjærsgaard et al., 2010; Buckley and Huey, 2016). We presume that the beetles might well use thermal information coded by bursty spike trains of the DSS neurons in the range of 35–45°C when suddenly confronted with these temperatures in their habitat. On an experimental thermal mosaic arena, the carabid beetle *P. assimilis* can easily discriminate between different levels of heat above 35°C (Merivee et al., 2015). In free-choice conditions, they spend substantially less time in areas with temperatures ranging from 40 to 45°C compared to those at 35–40°C. Further discriminatory experiments are needed, however, to specify how neuronal activity is correlated with functional discrimination of different levels of heat at rapid warming conditions.

Most insects need a sensitive thermo-sensory system to reliably sense ambient heat so as to avoid thermal stress. Besides antennal DSSs of carabids and elaterids, the classical sensory triad of thermo- and hygroreceptor neurons resides in various morphological types of antennal sensory organs, for example, in coeloconic sensilla widespread on the antennae in many insect taxa (Altner and Prillinger, 1980; Chapman, 1998; Ruchty et al., 2009; Schneider et al., 2018). The neurons of the sensory triad in coeloconic sensilla probably also use various spike burst patterns to encode ambient heat, but so far, this is not verified. Neither is data available on what the neural code is for heat detection in a great variety of thermosensory systems other than the classical sensory triad of thermo- and hygroreceptor neurons in the DSS sensilla of carabids and elaterids. Further studies are needed to shed the light on these questions.

## Data Availability Statement

The datasets generated during the current study are available from the corresponding author on reasonable request.

## Author Contributions

EM, AM, KN, and AD conceived and designed the experiments. EM, AM, and KN performed the physiological experiments. MMu and AD performed SEM images. EM, AM, KN, and MMä analyzed the data. EM, AD, and IW wrote the article. All authors interpreted the data and critically revised the manuscript.

## Conflict of Interest

The authors declare that the research was conducted in the absence of any commercial or financial relationships that could be construed as a potential conflict of interest.

## Footnotes

^1^http://www.ilmateenistus.ee

^2^http://www.ilmateenistus.ee
